# Effects of a comprehensive nursing intervention based on ERAS principles on postoperative outcomes in patients undergoing laparoscopic cholecystectomy: a randomized controlled trial

**DOI:** 10.3389/fsurg.2025.1629934

**Published:** 2025-11-17

**Authors:** Dingfeng Zhang, Ting Zhang

**Affiliations:** 1 Department of Nursing, Ankang People's Hospital, Ankang, Shaanxi, China; 2 Department of Nursing Headquarters, Ankang Central Hospital, Ankang, Shaanxi, China

**Keywords:** anxiety, complications, comprehensive nursing, depression, inflammatory response, laparoscopic cholecystectomy, pain, quality of life

## Abstract

**Objective:**

To assess the impacts of comprehensive nursing on pain score, negative mood along with quality of life in patients undergoing laparoscopic cholecystectomy (LC).

**Methods:**

Eighty-four patients (aged 40–65 years) who underwent LC in our hospital from March 2023 to March 2024 were chosen to be the study objects and were randomly separated into control group and study group. The control group accepted routine nursing. The study group accepted comprehensive nursing. The perioperative indicators, degree of pain, inflammatory response, anxiety and depression, sleep quality, incidence of postoperative complications, quality of life, as well as nursing satisfaction were compared in both groups.

**Results:**

The perioperative indicators of the study group were significantly better than those of the control group (*P* < 0.01), and the improvements in postoperative pain, inflammatory response, sleep quality, psychological state, and quality of life were more pronounced (all *P* < 0.05), with a lower incidence of complications (7.14% vs. 26.18%) and higher nursing satisfaction (95.24% vs. 76.19%).

**Conclusion:**

Comprehensive nursing can accelerate postoperative recovery, reduce the degree of pain, reduce inflammatory response, improve negative mood and sleep quality, decrease the incidence of postoperative complications along with promote the quality of life in patients undergoing LC. These findings suggest that comprehensive nursing should be considered as a standard care approach to optimize recovery for patients undergoing laparoscopic cholecystectomy.

## Introduction

Gallbladder diseases, primarily including cholelithiasis (gallstones), cholecystitis, and gallbladder polyps, represent a significant global health burden. The prevalence of gallstone disease, the most common biliary pathology, is estimated to be around 10%–15% in the adult population of developed countries, with variations based on geographic region, ethnicity, and diet. If not treated in a timely manner, these conditions can lead to serious complications such as acute cholecystitis, gallbladder empyema, gangrene, perforation, and secondary cholangitis, posing a substantial threat to patient health (1).

Recently, with the alteration of people's living and eating habits, coupled with the impact of life pressure, the clinical incidence of gallbladder disease has also shown a rising trend of development (2). Patients with gallbladder diseases are often accompanied by abdominal pain and fever and other symptoms, with the progress of the disease will also appear gallbladder swelling or suppurative cholangitis and other complications, threatening the life and health of patients (3). Currently, the primary treatment for symptomatic gallbladder diseases is surgery, with the main approaches being open cholecystectomy and laparoscopic cholecystectomy (LC) (4).

In recent years, with the rapid development of laparoscopic technology, LC has been widely applied and popularized in clinical practice due to its minimally invasive nature (5). LC has become the gold standard treatment for benign gallbladder conditions like gallstones and polyps (6). Relative to traditional open surgery, LC has the strengths of less trauma, high safety along with simple operation, which can effectively diminish the postoperative recovery time of patients, as well as promote the prognosis of patients (7). Therefore, it has been widely utilized in treating benign gallbladder diseases, and has played a definite clinical effect (8).

However, due to the lack of knowledge about LC and patients’ worries about operation, anesthesia and postoperative rehabilitation, patients are prone to different degrees of physiological and psychological stress, resulting in postoperative complications and unsatisfactory recovery effect, which affects the therapeutic effect. Therefore, implementing effective nursing measures is crucial to improving postoperative outcomes (9).

Comprehensive nursing is a patient-centered model integrating physiological, psychological, and rehabilitative interventions across perioperative phases. It emphasizes standardized procedures, multidisciplinary coordination, and individualized care plans, distinguishing itself from conventional nursing by addressing holistic patient needs (10). While conventional nursing often focuses primarily on postoperative monitoring and essential instructions, its effectiveness in improving patients’ mental health, pain management, and other nuanced aspects is limited. In contrast, comprehensive nursing, structured around the nursing process and combining the strengths of primary and team nursing, ensures coordination across all care aspects to enhance the level and quality of nursing services. It has been recognized as an effective model in various surgical departments (11). Nevertheless, robust evidence specifically quantifying its efficacy in mitigating LC-related psychological distress and modulating inflammatory responses remains relatively scarce.

Therefore, the purpose of our study was to assess the impacts of comprehensive nursing on pain score, negative mood, quality of life and other key postoperative outcomes in patients undergoing LC.

## Data and methods

### Sample size and study context

This study was designed as a prospective, randomized controlled trial. The sample size was not determined by a prior power calculation due to the exploratory nature of this intervention study and the constraint of a predefined one-year recruitment period (from March 2023 to March 2024). During this period, a total of approximately 150 patients underwent LC at our institution. Among them, 84 patients who met the inclusion criteria were enrolled and randomized. This sample size is comparable to those reported in similar studies evaluating nursing interventions for LC patients.

The nursing team involved in the study consisted of 25 registered nurses working in the general surgery department. The nurse-to-patient ratio was consistently maintained at 1:4 for both groups throughout the study period to control for workload bias. The average length of stay for LC patients in our department typically ranges from 3 to 7 days.

### General data

This study was designed as a prospective, randomized controlled trial. Eighty-four patients undergoing LC treated in our hospital from March 2023 to March 2024 were chosen to be the study objects and were randomly separated into control group and study group following the random number table method. Each group had 42 cases. Inclusion criteria: (1) The clinical diagnosis was benign gallbladder disease, and patients met the LC indication; (2) The patient's consciousness and cognition are normal. Exclusion criteria: (1) Patients complicated with choledocholithiasis, intrahepatic bile duct calculus, and obstructive jaundice; (2) Patients had malignant tumors; (3) Patients had serious heart, liver, kidney along with other viscera diseases. This study was approved by the Ethics Committee of the hospital with the informed consent of all patients.

### Surgical methods

LC was performed in both groups after admission, and pneumoperitoneum pressure of 12–14 mmHg was established after anesthesia. Laparoscopy and surgical equipment were implanted by three-hole method, gallbladder was removed under the laparoscopic field of view, Calot's triangle was dissected, proximal absorption was performed, distal closure was performed, gallbladder was removed, abdominal cavity was cleaned and sutured, and anti-infection treatment was performed after surgery.

### Nursing methods

The control group accepted routine nursing during the treatment. Routine nursing followed the hospital's standard postoperative care protocol: Preoperative: Fasting instructions (8 h for solids, 2 h for water) and basic surgical consent explanation. Intraoperative: Vital signs monitoring and sterile field maintenance. Postoperative: (1) Vital signs checks every 4 h for 24 h; (2) static bed rest until first flatus; (3) standard analgesic regimen (oral ibuprofen 400 mg q8h PRN); (4) discharge criteria: afebrile for 24 h, tolerating oral diet.

The study group implemented comprehensive nursing from various aspects according to the characteristics of the patients’ conditions. The comprehensive nursing model in this study was operationalized through four evidence-based components: (1) Structured patient education (preoperative); (2) protocolized pain management (intra-/postoperative); (3) psychological support cascade; (4) complication prevention bundles.

The details of comprehensive nursing were as follows:

1. Preoperative health education: Preoperative education focused on disease knowledge, treatment methods, surgical precautions and surgical necessity. According to the educational level and acceptance of patients, pictures, videos, books and other forms were selected to introduce them in detail, so that patients could understand their own disease characteristics and treatment methods, reduce their fear, and improve their cooperation with anesthesia and surgery.
2. Preoperative preparation: In terms of physiology, nurses helped the patient complete various examinations, instructed the family members to give the patient high-protein food to enhance nutrition before surgery, informed the patient to strictly refrain from drinking and fasting 1 day before surgery, and did a good job of surgical preparation such as cleaning, anesthesia and skin preparation to ensure the smooth operation. In terms of psychology, nurses communicated more with patients before surgery to develop a harmonious nurse-patient relationship, so as to facilitate the smooth development of nursing work. At the same time, nurses timely channeled the tension and fear of patients, so that patients could actively cooperate with preoperative examination and anesthesia.
3. Intraoperative comprehensive nursing: To ensure the comfort of patients from environmental, psychological and other aspects, the operating room was cleaned and disinfected following WHO surgical site infection prevention guidelines (chlorhexidine-alcohol solution, double-wipe technique) (12) in advance, prepared surgical instruments and drugs, maintained operating room at 21 ± 1°C and 50% ± 5% humidity per enhanced recovery after surgery (ERAS) protocols, helped patients to take positions during the operation followed ergonomic principles (15° reverse Trendelenburg for laparoscopic access; arm abduction <90°) to prevent brachial plexus injury, and timely established vein access. During the surgery, nurses focused on the thermal insulation nursing of patients, reduced cold stimulation such as rehydration and flushing solution, and maintained the comfort of patients through quilts and electric blankets if necessary. In addition, nurses reduced the skin exposure of unnecessary surgical areas and non-surgical areas were covered with sterile drapes, and only essential staff were permitted in the operating field.
4. Postoperative respiratory tract nursing: The establishment of artificial pneumoperitoneum will lead to the increase of internal abdominal pressure in patients, and the absorption of carbon dioxide in the abdominal cavity will easily lead to hypercapnia and respiratory acidosis. Nurses paid close attention to the patient's blood oxygen and other indicators, kept the patient's respiratory tract unobtrusive, and gave patients continuous or intermittent oxygen inhalation to promote the discharge of carbon dioxide.
5. Postoperative pain nursing: Nurses gave the patient pain relief pump 12–48 hours after surgery and then guided the patient to relieve the pain by shifting attention, regulating breathing, and playing soothing music, and trained nurses performed standardized effleurage massage (10 min sessions, 2× /day) on paravertebral muscles using WHO-recommended techniques, thus relieving the pain.
6. Postoperative comprehensive diet guidance: After surgery, the patient was instructed to chew gum to promote gastrointestinal peristalsis and instructed to focus on liquid and semi-liquid foods to ensure normal intestinal peristalsis. The patient refrained from gas producing foods, such as radish and soybean products. Within 7 days after surgery. The postoperative diet of patients was low-fat, high-protein and high-calorie, with light and easily digestible foods and frequent meals. Patients avoided eating too much and eating spicy, cold and greasy food.
7. Postoperative complications nursing: After the patient was awake, nurses assisted the patient to do bed activities to speed up blood circulation, and the patient could conduct semi-decumbent position and get out of bed activities in the early stage to prevent venous thrombosis and promote recovery. Nurses paid close attention to the occurrence of complications of the patient, ensured that the urinary catheter was patent, avoid infection of the patient. Besides, nurses accurately assessed the patient's subcutaneous emphysema, performed oxygen intervention, changed the incision in time, and kept the incision dry and clean. If the patient was found to have bile leakage, the drainage tube was placed in time. Once the patient had abdominal pain, fever, and bile-like fluid, nurses timely reported to the doctor and corrected treatment to reduce the risk of complications.
8. Psychological nursing: Before operation, nurses established a good nurse-patient relationship with patients, strengthened communication, understood the causes of patients’ anxiety and fear, corrected cognition through health education in time, and gave targeted intervention and guidance, and balanced patients’ preoperative mentality. After surgery, for patients with anxiety such as pain, nurses instructed them to divert their attention by chatting with family members, listening to music, and watching videos, and encouraged family members to accompany patients, so that patients could obtain spiritual support and strengthen the recovery of confidence.

Both groups received equal nursing staffing ratios (1 nurse: 4 patients) to control for workload bias. The nursing care was delivered by a dedicated team of 25 registered nurses. All held a bachelor's degree or higher, with a mean age of 32.5 ± 4.2 years and a mean professional experience of 8.5 ± 3.1 years. This team had received standardized training in the comprehensive nursing protocol based on ERAS principles prior to the study commencement to ensure consistency in intervention delivery.

### Observation indicators

1. Perioperative indicators including extraction time, feeding time, exhaust time, bowel sound recovery time as well as hospital stay were recorded in both groups.
2. Visual analogue scale (VAS) was employed for evaluating the degree of pain (13), with a total of 10 points, and the higher the score, the more severe of the pain.
3. Three ml fasting venous blood was extracted from the patient. After serum separation, the enzyme-linked immunosorbent assay were employed for detecting the serum levels of tumor necrosis factor-α (TNF-α) as well as interleukin-6 (IL-6).
4. Self-rating Anxiety Scale (SAS) and Self-rating Depression Scale (SDS) were employed for evaluating patients’ anxiety and depression (14). SAS score >50 was anxiety, SDS score >53 was depression, and the higher the score was, the more serious the anxiety and depression was, respectively.
5. Pittsburgh Sleep Quality Index (PSQI) was employed for assessing patients’ sleep quality (15). The total score was 21 points, the higher the score, the worse the sleep quality.
6. Incidence of postoperative complications including bile leakage, intraperitoneal hemorrhage, infection and pneumoderm was recorded in both groups.
7. Generic Quality of Life Inventory-74 (GQOLI-74) was employed for evaluating patients’ quality of life (16), which included material life, physical function, mental function along with social function, with scores ranging 0–100. The score was directly proportional to quality of life.
8. After the end of nursing, nursing satisfaction questionnaire made by our hospital was employed to assess the contents, including service enthusiasm and initiative, work ability, care and communication, ward management, and health education. Score >90 was very satisfied, 70–90 was satisfied, <70 was dissatisfied. Satisfaction = (number of very satisfied cases + number of satisfied cases)/total number of cases ×100%.

### Statistical analysis

Statistical analysis was performed using SPSS software (version 25.0; IBM Corp., Armonk, NY, USA). Continuous data with normal distribution are presented as mean ± standard deviation (x ± s) and were compared between groups using the independent samples *t*-test. Categorical data are presented as number (percentage) and were compared using the Chi-square (*χ*^2^) test or Fisher's exact test as appropriate. The sample size was not determined by a formal power calculation but was based on the number of eligible patients available during the one-year study period and aligned with sample sizes reported in comparable studies of nursing interventions. A two-tailed *P*-value of <0.05 was considered statistically significant for all analysis.

### Bias control

To minimize potential bias, patients were randomly allocated using a computer-generated random number table. The outcome assessors were blinded to the group assignment. All nursing interventions were carried out following standardized protocols to ensure consistency. Furthermore, the nurse-to-patient ratio was maintained equally between both groups.

## Results

### Baseline characteristics and nurse qualifications

As detailed in Table 1, the two groups were well-matched at baseline. There were no statistically significant differences in demographic characteristics (age, gender), clinical profiles (types of gallbladder disease, prevalence of hypertension or diabetes mellitus), or surgical history (previous abdominal surgery). All patients in both groups received the same balanced intravenous-inhalation anesthetic technique. Furthermore, as outlined in the table, the qualifications of the nursing staff responsible for both groups were equivalent, ensuring that any observed differences in outcomes could be attributed to the nursing intervention itself rather than to disparities in patient baseline status or nursing expertise.

**Table 1:** Comparison of general data and baseline characteristics between the two groups.

Characteristics	Control group (*n* = 42)	Study group (*n* = 42)	t/*χ*^2^ value	*P*-value
Demographics				
Age (years, $\bar{x}\pm s$)	42.85 ± 8.43	42.92 ± 8.51	0.03	0.96
Gender (Male/Female)	26 (61.90%)/16 (38.10%)	25 (59.52%)/17 (40.48%)	0.04	0.82
Disease type				
Cholelithiasis	18 (42.86%)	19 (45.24%)	0.22	0.89
Cholecystitis	10 (23.81%)	11 (26.19%)		
Gallbladder polyps	14 (33.33%)	12 (28.57%)		
Comorbidities				
Hypertension	8 (19.05%)	7 (16.67%)	0.08	0.78
Diabetes Mellitus	5 (11.90%)	4 (9.52%)	0.13	0.72
Surgical history				
Previous abdominal surgery	6 (14.29%)	5 (11.90%)	0.10	0.75
Anesthetic technique				
Intravenous-inhalation balanced	42 (100%)	42 (100%)	N/A	N/A
Nurse qualifications	(*n* = 25)	(*n* = 25)		
Education (Bachelor's degree or above)	100%	100%	N/A	N/A
Age (years, $\bar{x}\pm s$)	32.5 ± 4.2	32.5 ± 4.2	N/A	N/A
Years of professional experience ($\bar{x}\pm s$)	8.5 ± 3.1	8.5 ± 3.1	N/A	N/A

### Perioperative indicators in both groups

**Figure 1:**
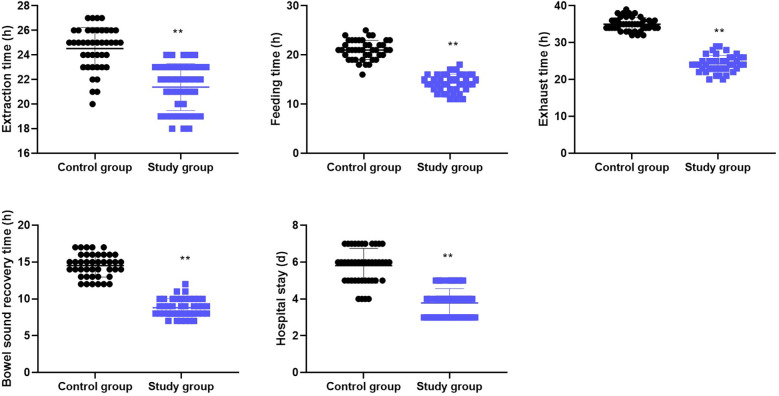
Perioperative indicators in both groups. Data are presented as mean ± standard deviation. **p < 0.001, indicates a statistically significant difference between the control and study groups for all indicators (independent samples *t*-test).

As shown in Figure 1, the study group presented significantly shorter extraction time, feeding time, exhaust time, bowel sound recovery time, and hospital stay compared to the control group (all *P* < 0.001).

### Degree of pain in both groups

Before nursing, the VAS score showed no difference between the two groups. Following nursing, the VAS score declined significantly in both groups (*P* < 0.001), and the score in the study group was significantly lower than that in the control group (*P* < 0.001, Table 2).

**Table 2:** Comparison of secondary outcomes: pain, inflammatory response, psychological status, sleep quality, and quality of life between the two groups (mean ± SD).

Outcome measure	Group	Before nursing	After nursing	*P*-value (within group)	*P*-value (between groups, post-nursing)
Pain (VAS score)	Control	6.8 ± 1.1	3.2 ± 0.7	<0.001	<0.001
	Study	6.7 ± 1.0	1.9 ± 0.5	<0.001	
Inflammatory markers					
TNF-α (pg/mL)	Control	45.2 ± 8.1	28.5 ± 5.3	<0.001	<0.001
	Study	44.8 ± 7.9	20.1 ± 4.1	<0.001	
IL-6 (pg/mL)	Control	32.5 ± 6.4	19.8 ± 4.2	<0.001	<0.001
	Study	33.1 ± 6.0	14.3 ± 3.5	<0.001	
Psychological status & sleep					
SAS score	Control	58.4 ± 6.2	46.3 ± 5.1	<0.001	<0.001
	Study	57.9 ± 5.8	38.5 ± 4.4	<0.001	
SDS score	Control	56.8 ± 5.9	45.1 ± 4.8	<0.001	<0.001
	Study	57.2 ± 5.5	37.2 ± 4.0	<0.001	
PSQI score	Control	12.5 ± 2.3	8.8 ± 1.7	<0.001	<0.001
	Study	12.3 ± 2.1	6.1 ± 1.3	<0.001	
Quality of life (GQOLI-74)					
Physical function	Control	65.2 ± 7.1	72.5 ± 6.3	<0.001	<0.001
	Study	64.8 ± 6.8	81.3 ± 5.9	<0.001	
Psychological function	Control	62.1 ± 6.5	70.8 ± 5.8	<0.001	<0.001
	Study	61.7 ± 6.2	79.5 ± 5.5	<0.001	
Social function	Control	66.5 ± 6.9	73.9 ± 6.0	<0.001	<0.001
	Study	66.0 ± 6.6	82.1 ± 5.8	<0.001	
Material life	Control	68.8 ± 7.3	72.1 ± 6.5	0.005	0.002
	Study	69.1 ± 7.0	76.5 ± 6.2	<0.001	

VAS, visual analogue scale; TNF-α, tumor necrosis factor-alpha; IL-6, interleukin-6; SAS, self-rating anxiety scale; SDS, self-rating depression scale; PSQI, Pittsburgh sleep quality index; GQOLI-74, generic quality of life inventory-74.

*P*-value (Within Group): Comparison before vs. after nursing within the same group (paired *t*-test). *P*-value (Between Groups): Comparison between the control and study groups after nursing (independent samples *t*-test).

### Serum levels of inflammatory mediators in both groups

Before nursing, the serum levels of inflammatory mediators showed no difference between the two groups. Following nursing, the serum levels of TNF-α and IL-6 declined significantly in both groups, and the levels in the study group were significantly lower than those in the control group (*P* < 0.001 for both, Table 2).

### Anxiety and depression in both groups

Prior to nursing, the SAS and SDS scores showed no difference between the two groups. Following nursing, the SAS and SDS scores declined significantly in both groups (*P* < 0.001), and the scores in the study group were significantly lower than those in the control group (*P* < 0.001, Table 2).

### Sleep quality in both groups

Prior to nursing, the PSQI score showed no difference between the two groups. Following nursing, the PSQI score declined significantly in both groups (*P* < 0.001), and the score in the study group was significantly lower than that in the control group (*P* < 0.001, Table 2).

### Quality of life in both groups

Before nursing, the GQOLI-74 scores showed no difference between the two groups. Following nursing, the GQOLI-74 scores across all domains were elevated in both groups, and the scores in the study group were significantly higher than those in the control group (all *P* < 0.01, Table 2).

### Incidence of postoperative complications in both groups

In comparison with the control group (11/42, 26.18%), the study group presented lower incidence of postoperative complications (3/42, 7.14%) (*P* = 0.018, Table 3).

**Table 3:** Incidence of postoperative complications in both groups.

Groups	Cases	Bile leakage	Intraperitoneal hemorrhage	Infection	Pneumoderm	Total incidence rate
Control group	42	3 (7.14)	2 (4.76)	3 (7.14)	3 (7.14)	11 (26.18)
Study group	42	1 (2.38)	0 (0.00)	1 (2.38)	1 (2.38)	3 (7.14)
χ^2^ value						5.48
*P* value						0.01

### Nursing satisfaction in both groups

In comparison with the control group (32/42, 76.19%), the study group presented higher nursing satisfaction (40/42, 95.42%) (*P* = 0.013, Table 4).

**Table 4:** Nursing satisfaction in both groups.

Groups	Cases	Very satisfied	Satisfied	Dissatisfied	Total satisfaction rate
Control group	42	15 (35.71)	17 (40.48)	10 (23.81)	32 (76.19)
Study group	42	20 (47.62)	20 (47.62)	2 (4.76)	40 (95.24)
χ^2^ value					6.22
*P* value					0.01

## Discussion

This randomized controlled trial demonstrates that a comprehensive nursing intervention grounded in ERAS principles significantly enhances postoperative recovery for patients undergoing LC. Specifically, the intervention group exhibited markedly accelerated recovery of gastrointestinal function and shorter hospitalization, attenuated postoperative pain and systemic inflammatory response, improved psychological well-being and sleep quality, and ultimately, a lower incidence of complications and higher quality of life coupled with greater patient satisfaction.

The significant reduction in time to first exhaust, bowel sound recovery, feeding, and ultimately, hospital stay, in the study group underscores the efficacy of the comprehensive nursing model in promoting rapid recovery. We postulate that this synergistic effect stems from multiple components of the intervention: preoperative chewing gum to stimulate gastrointestinal peristalsis, structured early dietary advancement with gas-reducing and easily digestible foods to minimize burden, and early ambulation encouraged by proactive nursing support. These measures collectively mitigate postoperative ileus, a common driver of prolonged hospitalization. This finding aligns with the core objectives of ERAS protocols and is consistent with studies in other surgical contexts, such as the work by Yu et al. in colostomy patients, which reported enhanced recovery with comprehensive care (17).

Our study provides compelling evidence that the comprehensive nursing intervention effectively alleviates both subjective pain (VAS scores) and objective biochemical inflammation (TNF-α, IL-6). This dual effect is critically important. We hypothesize that the protocolized pain management—combining timely analgesic administration (pain relief pump) with non-pharmacological techniques (distraction, effleurage massage)—directly reduces nociceptive stimulation. This, in turn, likely blunts the surgical stress response, leading to a downstream reduction in the production of pro-inflammatory cytokines like IL-6 and TNF-α. This mechanistic insight strengthens the biological plausibility of our findings and finds support in other specialties; for instance, Zhong et al. suggested that comprehensive nursing intervention effectively reduced the degree of pain in patients with rupture and bleeding of ectopic pregnancy (18); Sun et al. suggested that comprehensive care in knee osteoarthritis improved pain, potentially via reducing inflammatory mediator (19).

The significant improvements in SAS, SDS, and PSQI scores in the intervention group highlight the profound impact of addressing patients’ psychological needs. The sustained psychological support cascade, from preoperative education that manages expectations to postoperative strategies for anxiety and distraction, provides continuous emotional regulation. This holistic approach likely reduces preoperative fear and postoperative distress, which are known to negatively impact sleep architecture and pain perception. The observed correlation between lower anxiety scores and reduced inflammation (e.g., lower IL-6) in our study supports the well-established bi-directional communication between the psychological state and inflammatory pathways (20). Our results corroborate the findings of Chen et al., who demonstrated that comprehensive nursing alleviated negative emotions in patients undergoing peritoneal dialysis catheter insertion (21).

The >70% relative reduction in total complications (26.18%–7.14%) is a clinically paramount outcome. This can be directly attributed to the proactive complication prevention bundles integral to our intervention: vigilant monitoring for bile leakage and hemorrhage, meticulous respiratory care to prevent atelectasis and hypercapnia, and stringent infection control measures. By preventing these setbacks, the intervention safely facilitates early recovery and discharge. Consequently, patients experienced a superior quality of life across all domains and reported significantly higher satisfaction with their care. Li et al. pointed out that comprehensive nursing improved the quality of life of prostate cancer patients undergoing chemoradiotherapy, reduced their adverse reactions (22). This holistic improvement in patient-reported outcomes and safety profile is the ultimate goal of nursing care and resonates with studies like that of Xiang et al. in spinal cord injury patients (23).

The main strength of this study lies in its robust prospective randomized design and the multi-faceted, evidence-based nature of the nursing intervention, which was delivered by a standardized nursing team. Nevertheless, several limitations must be acknowledged. Nevertheless, several limitations must be acknowledged. First, the single-center design and the specific demographic characteristics of our sample may affect the generalizability of our findings to other populations or healthcare settings. Second, while outcome assessors were blinded, the nature of the complex nursing intervention made it impossible to blind the patients and nursing staff, which introduces a potential for performance bias. Third, the absence of a formal sample size calculation *a priori*, as previously discussed, is a methodological limitation, though our *post-hoc* analysis confirms the observed differences were highly statistically significant. Finally, the lack of long-term follow-up data precludes any conclusions about the sustainability of the observed benefits beyond the immediate postoperative period.

Future research should address these limitations through multicenter trials with larger, more diverse populations and long-term follow-up to assess the durability of outcomes. Furthermore, cost-effectiveness analyses are warranted to evaluate the economic implications of implementing this labor-intensive model, and mechanistic studies exploring the interplay between psychological support, stress biomarkers, and clinical outcomes would be invaluable.

In conclusion, our findings strongly suggest that the implementation of a comprehensive, ERAS-based nursing intervention is a highly effective strategy to optimize the entire care pathway for LC patients. The quantifiable benefits—a 19.04% absolute reduction in complications and a 1.7-day shorter average hospital stay—translate not only into improved patient well-being but also into potential significant healthcare cost savings. Therefore, we recommend that healthcare institutions consider the adoption and integration of such structured comprehensive nursing protocols into standard clinical practice for patients undergoing laparoscopic cholecystectomy.

## Data Availability

The datasets presented in this study can be found in online repositories. The names of the repository/repositories and accession number(s) can be found in the article/Supplementary Material.
